# sEMG and Vibration System Monitoring for Differential Diagnosis in Temporomandibular Joint Disorders

**DOI:** 10.3390/s22103811

**Published:** 2022-05-17

**Authors:** Małgorzata Kulesa-Mrowiecka, Robert Barański, Maciej Kłaczyński

**Affiliations:** 1 Department of Rehabilitation in Internal Diseases, Institute of Physiotherapy, Faculty of Health Science, Jagiellonian University Medical College, Skawińska Str. 8, 31-066 Krakow, Poland; 2 Department of Mechanics and Vibroacoustics, Faculty of Mechanical Engineering and Robotics, AGH University of Science and Technology, al. A. Mickiewicza 30, 30-059 Krakow, Poland; robert.baranski@agh.edu.pl (R.B.); maciej.klaczynski@agh.edu.pl (M.K.)

**Keywords:** sEMG, vibration, health monitoring, biomedical sensors, rehabilitation technology, telemedicine, stomatognathic physiotherapy

## Abstract

The stomatognathic system represents an important element of human physiology, constituting a part of the digestive, respiratory, and sensory systems. One of the signs of temporomandibular joint disorders (TMD) can be the formation of vibroacoustic and electromyographic (sEMG) phenomena. The aim of the study was to evaluate the effectiveness of temporomandibular joint rehabilitation in patients suffering from locking of the temporomandibular joint (TMJ) articular disc by analysis of vibrations, sEMG registration of masseter muscles, and hypertension of masticatory muscles. In this paper, a new system for the diagnosis of TMD during rehabilitation is proposed, based on the use of vibration and sEMG signals. The operation of the system was illustrated in a case study, a 27-year-old woman with articular dysfunction of the TMJ. The first results of TMD diagnostics using the k-nearest neighbors method are also presented on a group of fifteen people (ten women and five men). Vibroacoustic registration of temporomandibular joints, sEMG registration of masseter muscles, and functional manual analysis of the TMJ were simultaneously assessed before employing splint therapy with stomatognathic physiotherapy. Analysis of vibrations with the monitoring of sEMG in dysfunctions of the TMJ can lead to improve differential diagnosis and can be an objective way of monitoring the rehabilitation process of TMD.

## 1. Introduction

**Figure 1:**
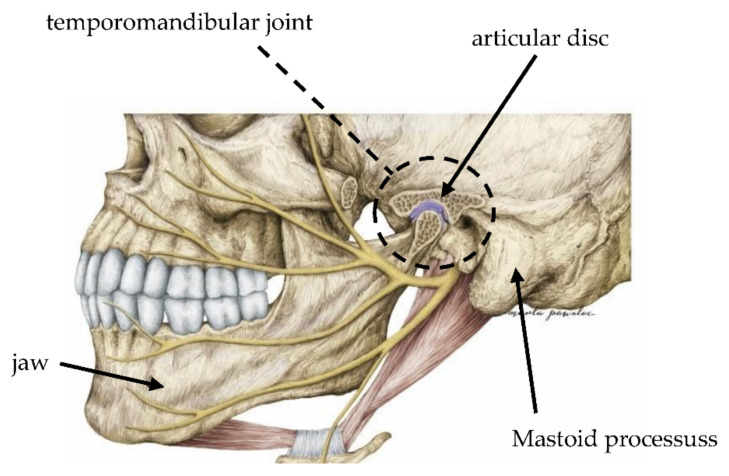
Anatomy of the area of temporomandibular joint (TMJ).

The temporomandibular joints (TMJ) are the 2 joints that connect the lower jaw to the skull (Figure 1). Patients with temporomandibular disorders (TMD) are characterized by the experience of pain, limited or excessive mobility of the temporomandibular joints (TMJ), mandible deviation, or acoustic sensations. One of the symptoms of TMD is the formation of vibroacoustic phenomena [1]. As many as 10–40% of people aged over 18 experience pain due to TMD. The number of patients with morbidity related to the TMJ and the facial, head, and neck area is increasing in modern society [2]. Disturbances in this region, including acoustic sensations of the body, may have a psychosocial dimension and affect the self-esteem of patients [3]. Most often these are sounds of transitional transformations or results of parafunctions resulting from clicking, popping, friction, or crepitation. Acoustic sensations are quite common, but do not always require treatment [4,5]. The causes of vibroacoustic signs within the temporomandibular joints are complex, but they always involve the loss of coordination between the disc and the condyle during their movements and the change in tension on ligaments and muscles. The most common cause of this dysfunction and these symptoms is bruxism [6,7,8,9,10].

The central position of the TMJ articular surfaces is maintained by the activity of muscle tone, proper tension of the ligaments, and the joint capsule [11,12].

When the articular disc is locking, the characteristic symptoms and signs include pain, restriction of mobility, disturbance of joint function, and acoustic sensations [13]. When the articular disc is unlocked, characteristic cracking sounds may be heard during patient examination. Often, a double crack occurs: the first one during mandibular abduction and another reciprocal click occurs when the mouth is closed just before teeth occlusion [2,14]. Sounds noted around the TMJ are named as acoustic phenomena (AP). AP occur during the displacement of the disc but also in developed changes in the TMJ and have different characteristics than crepitations. Most often, anterior dislocations occur; however, rarely may medial and posterior dislocations occur. The following progression is observed in TMJ pathologies: sporadic clicks in the irregular displacement of the articular disc, cracks in the dislocation of the disc without blocking, abnormal path of movement of the mandible, blocking of the disc, degenerative changes of the joint, and frequent crepitations caused by friction. TMD can occur unilaterally or bilaterally. TMDs are connected to both joints if one has changes to its compression mechanism [15].

The first attempts to use vibroacoustic signals as a carrier of diagnostic information for the dysfunction of the temporomandibular joints took place in the second half of the 20th century. This research focused on characterizing the acoustic signals generated in healthy joints and those with motor disorders [16,17]. The sound pressure levels accompanying the dysfunction phenomena were determined, as well as the duration of characteristic clicks [18,19]. Subsequent associations were drawn between the presence of significant vibrational energy within the examined joint and the degree of changes. Vibrations of the highest amplitude and frequency from 8.50 to 57.61 Hz were observed in the middle phase of the mandibular abduction cycle in people with joint dysfunction [20]. Some researchers revealed (in a group of 138 patients) imperfections in the use of electrovibrography (EVG) due to the ambiguity in the interpretation of the signals generated by the joints. Vibrations in the temporomandibular joint were usually associated with disturbances inside the joint; however, there were cases of recording an acoustic wave also in a healthy joint [21]. These reports are opposed by studies that show that a significant proportion of properly functioning joints did not cause vibrations of such high amplitude and frequency as in the case of affected joints.

Several methods are used to diagnose TMD (acoustic signals [18,19], electrovibrography [21], and magnetic resonance imaging [15]), but still clinicians and researchers do not agree whether the analysis of sounds of the TMJ is sufficient for a proper differential diagnosis of TMD [14,15,22,23,24]. The current state of the art criteria for the diagnosis of TMD is presented in the following part of this section.

The authors’ approach presented in this paper was to analyze the vibrations generated in the temporomandibular joints during their movement, along with the simultaneous measurement of sEMG signals and video activity. In particular, the parameterization of the vibration signal to a feature vector in correlation with the analysis and parameterization of the sEMG signal are proposed in this paper. A measurement system was created and example results during rehabilitation with a patient (a 27-year-old woman) suffering from articular dysfunction of the TMJ are presented. Results of the first attempts to use the k-nearest neighbors (k-NN) method to test the effectiveness of assigning sick and healthy patients are also presented. Such an approach to TMD has not yet been under consideration and published.

### Temporomandibular Disorder Diagnostics

There are a number of studies that showed the possibility of using vibration analysis in diagnosing disorders of the temporomandibular joints [25,26,27,28]. Kłaczyński’s study [1] showed a greater usefulness of recording the vibration signal (three directional components for each joint) compared to the recording of the acoustic signal itself using various recording techniques: external microphones, ear microphones, or stethoscope. To confirm the usefulness of electrovibrography as a complementary tool to the pivotal patient examination, the results were compared with standard methods that gave measurable results. The effectiveness of the EVG test in detecting the two most common abnormalities of the temporomandibular joint has also been demonstrated in different cases: dislocation of the articular disc with and without reduction. These are problems manifested by audible clicks in the various phases of mandibular abduction and adduction. The form of the acoustic signal corresponding to the movement of the joint with the displacement of the disc turned out to be easily distinguishable from the vibration wave generated in a healthy joint [29]. Further development of the research extended the possibilities of vibration analysis to identifying a specific disorder type based on the corresponding characteristic frequency of vibrations [23]. The distinctive shape of the acoustic wave was also distinguished in some patients with exudative arthritis. The fact that this type of disorder requires magnetic resonance imaging (MRI) in order to make a certain diagnosis is of significant importance [30]. Not all attempts to demonstrate the effectiveness of EVG confirmed its usefulness in the diagnosis of a given disease. In one of the studies, the results of vibration analysis were compared with the results of the MRIs of patients with flattening of the articular tubercle or deepening of the fovea as a result of the natural adaptation to the changes, and of patients with a damaged surface of the condylar processes. EVG has shown that joints with adapted surfaces do not generate high frequencies of vibration, which makes it extremely difficult to make a correct diagnosis [31]. Vibration analysis makes it possible to identify the presence of a disturbance in the internal structure of the joint on the basis of acoustic symptoms, but it does not allow for the detection of its exact cause. Assessment of the structure and diagnosis of morphological defects of the temporomandibular joint are only possible with the use of MRI and a computed tomography (CT). However, it has been shown that supplementing the patient examination with EVG would allow for, in some cases, the omission of these complex and expensive techniques, for faster diagnosis and a significant reduction in the time needed to start treatment [32]. Other works have reaffirmed the methods of EVG in diagnosing TMD [33,34,35,36].

The search for methods of objective evaluation of the function of the temporomandibular joints has shown the usefulness of a completely different technique, surface electromyography (sEMG), i.e., the study of the electrical activity of the muscles responsible for the work of these joints [37,38,39,40]. Santana-Mora et al. [37] assessed the differences in electromyographic activity recorded during locking in women with chronic unilateral TMD compared to control patients. It has been proven that the asymmetry index (AI) can be a useful measure of differentiating right-sided and left-sided TMD patients. A preliminary study [38] showed that sEMG highlighted the differences in the activation time of muscles; thus, people with TMD showed neuromuscular changes that can lead to functional changes. Subsequent studies concerned the study of the difference in absolute EMG values for the healthy group and the TMD group before and after wearing braces and the change in the location of the temporomandibular joint using an X-ray imaging method and 3D face photography [40].

## 2. Materials and Methods

The following described measurement system was tested during measurement sessions in fifteen patients. All subjects underwent an identical measurement process.

The patient was examined in a physiologically correct sitting position, controlled by a physiotherapist with over 20 years of experience. The order of exercises performed in the full range of motion was decided. In the initial phase of the study, an attempt was made to record the snap of the joints.

- Three-click taps used to synchronize signals;
- Slow opening (SO)—three times;
- Fast opening (FO)—three times in each direction;
- Fast adduction (FA)—three times;
- Opening with protruding (OP) the mandible and returning to the rest position;
- Slow protruding (SP)—three times;
- Translation to the right (TR)—slow movements from side to side;
- Translation to the left (TL)—slow movements from side to side;
- Clenching without pads (CWP)—5 s of contraction and 10 s of rest;
- Clenching with pads (CP)—5 s of contraction and 10 s of rest;
- Stopping in the rest position and choking (CH)—three times in each direction;
- Slow coordination tongue exercises (TE)—three times.

**Figure 2:**
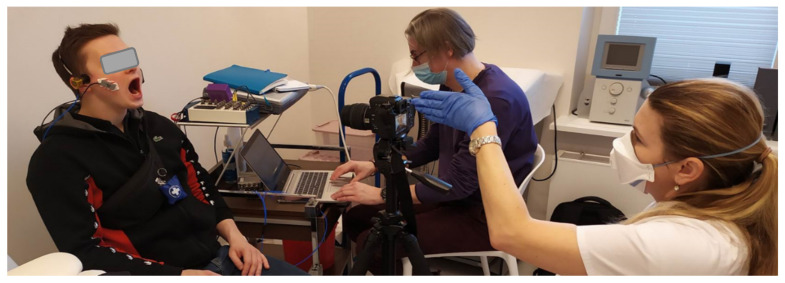
Examination of the patient with TMD—an overview.

**Figure 3:**
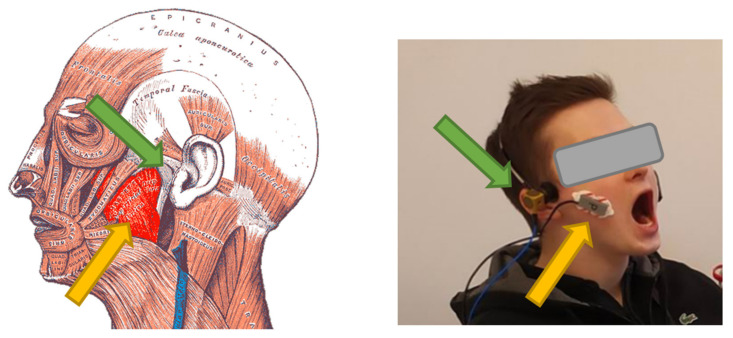
Examination of the patient with TMD. Indication of EMG electrode mounting location (yellow arrow) and acceleration sensor mounting location (green arrow). (Anatomical image from Wikipedia under public domain rights).

We decided to use the Pentax K5 camera to record the video of the patient’s face (Figure 2). This camera was selected in a previous study [1]. The main reason for using the camera was to record information about the start and end of the movement. We also planned to use videos to calculate range of motion as well as collect information on whether extra muscles were involved during exercise, such as the tongue or orbicularis oris. The recording also informs whether the patient has physiological limitations (Figure 3); in this type of disease, such limitations are not uncommon. To handle video files and their synchronization with sEMG and the vibration signals, a REAPER—Digital Audio Workstation was used. The video recordings were synchronized with the vibration course due to the fact that each exercise repeated by the patient began with tapping the teeth against each other three times. On the other hand, sEMG signals were recorded synchronously with the vibrations course; —this was ensured by the measurement system (National Instruments measurement cards and software developed in LabVIEW environment).

For the sEMG signal, we focused on measuring masseter muscle activity occurring on both sides of the head (Figure 3). This muscle consists of two layers—the superficial layer and the deep layer. The location of the electrodes was determined by palpating the muscle belly (superficial layer), which is most easily activated by asking the patient to clench their teeth. Once the belly site was determined, the patient’s skin was cleansed of keratinized epidermis and then washed with disinfectant (all women were asked to remove makeup prior to the study).

The study was conducted with the patient in a sitting position to simulate proper movement of the jaw while speaking, eating, expressing emotions, and for proper tension of the tongue, suprahyoid, and infrahyoid muscles. The patient was instructed to sit upright on a chair with the unsupported head looking forward in a horizontal position, both feet on the floor, hands resting on the lap. A vibration accelerometer was mounted on a specially prepared headband and placed 5 mm in front of the external ear canal. The method of assembly and the choice of sound recording technique were a previous subject of research [1].

The sEMG device was placed according to the technique described by Ferrario and Sforza [41], by placing it over the belly of the muscle in the largest area with regard to the amount of the neuromuscular junctions (Figure 3). Before placing, the skin was cleaned with 95% alcohol. The electrodes were placed bilaterally on the subject’s skin, perpendicular to the skin surface, parallel to muscular fibers, with the upper pole of the electrode at the intersection between the tragus–labial commissure and the exocanthion–gonion lines [41,42,43,44,45], but with modifications of the placement of the reference electrode, which was attached to the styloid process of the ulna instead of the forehead to not disturb the measurements of the vibration accelerometer. Dual pre-gel disposable electrodes (Ag/AgCl) were used to measure the EMG signal with a center-to-center distance of 2 cm and a pre-gel diameter of 14 mm. The electrodes were fixed on the forearm in the places shown in Figure 3. The reference electrode was attached to the coronoid process of the ulna.

During the implementation of previous studies [1], very good results in the reproduction of the vibration signal during testing were obtained using a headband from standard headphones providing a sensor contact force in the range of 2.5–2.8 N (since the headband is an elastic element, from a mechanical point of view it represents a spring, which for different head sizes will exert a different contact force).

This level of force ensured patient comfort during the test and did not interfere with joint function.

In Section 3 of this article, the results of the analysis of two different approaches to the analysis of the recorded signals are presented. One is the analysis of the progress of rehabilitation of one patient, while the second case is an attempt to recognize the state of the subject (healthy or unhealthy) using the currently recorded database of signals and the k-nearest neighbors (k-NN) method. For both cases, the data acquisition process was identical (the same equipment, sitting position, number of exercises, procedure, and measuring apparatuses).

### 2.1. Patient in the Rehabilitation Process

This article includes the presentation of a complete one-patient study of a Caucasian 27-year-old woman suffering from articular dysfunction based on the diagnostic criteria or research diagnostic criteria (DC RDC) classification of TMD: a patient with disc displacement with a reduction in the left TMJ and co-occurred myofascial pain of masseter muscles [41]. Vibroacoustic registration of temporomandibular joints, sEMG registration of masseter muscles, and functional manual analysis of the TMJ were assessed before splint therapy with stomatognathic physiotherapy. Functional manual analysis assesses the range of motion of the jaw, pattern of jaw movements, a group of crackles, myofascial pain (visual analogue scale), trigger points, and posture assessment. Effects on the change in the range of motion were assessed and the scale of dysfunction according to the DC of TMD and pain according to the numeric pain rating scale (NPRS) were evaluated. Examination and manual therapy were aimed at activating the articular capsule, reducing intra-articular pressure, and improving the range of mandibular mobility. Physiotherapeutic procedures, myofascial therapy, soft tissue therapy, and therapy of trigger points were performed.

After examination, the patient underwent splint therapy and physiotherapy according to the recommended Integrated Approach to Craniomandibular Muscle Exercises [14]. Splint therapy involved a relaxing nightguard used by the patient for 21 h/24 h during first 3 months, then 8 h/24 h during 6–12 months of observation. Muscle exercises were performed to improve gloss–mandibular coordination. When the deviation of the jaw was to the left, the position of the tongue was found to be on the right side to attain the proper kinesiology of the jaw in the middle and to eliminate the crepitations or clicking in the TMJ. The authors included an individual set of exercises, selected depending on the patient’s malocclusion in overbite position of the tongue anteriorly or underbite position of the tongue posteriorly. These exercises were recommended as at-home therapy consisting of 10–20 repetitions 3 times a day. The follow-up measurements were assessed 3 months and 1 year after therapy.

### 2.2. Acquisition System

**Figure 4:**
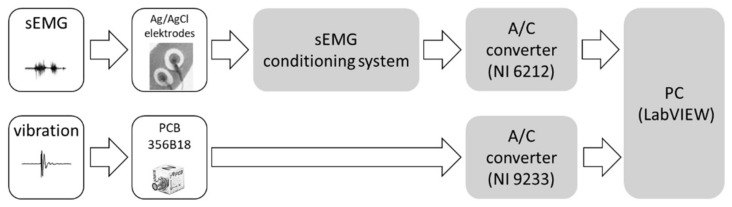
Block diagram of the measuring system.

In our system, two different types of commonly known diagnostic signals were used: vibrations and sEMG. All signals were recorded in the same way for the left and right side of the patient’s face by a designed system—hardware and software. For vibration measurement, a triaxial accelerometer PCB model 356B18 (precision, high sensitivity: 1000 mV/g, frequency range: 0.5 to 3 k Hz) connected to analog-digital (A/D) converter NI USB 9233 (4 channel, ±5 V, 24-bit A/C) was used. For sEMG signal measurement, a conditioner of our own production with A/D converter NI USB-6212 BNC (16 channel, ±10 V, 16-bit A/C) was used. The sEMG conditioner had been designed in accordance with the SENIAM guidelines (Surface Electromyography for the Non-Invasive Assessment of Muscles) [42]. Based on the guidelines, the INA 128P precision amplifier was used (CMRR > 100 dB), with gain 1200 and analog filter (Sallen-Key topology) with a range of 6 Hz ÷ 1000 Hz [43]. All vibration and sEMG signals were sampled with a frequency of 10 kHz. A block diagram of the measuring system is shown in Figure 4.

**Figure 5:**
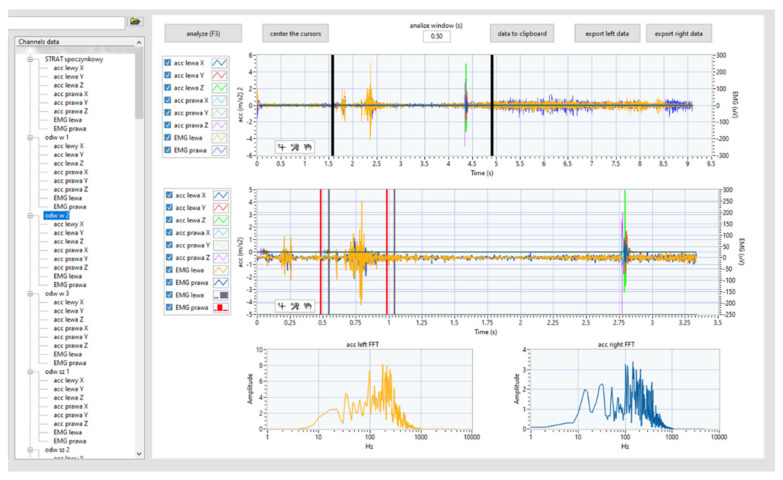
The analyzing system—front panel.

A dedicated software written in LabVIEW 2019 was used to collect and process all measured data. The program was dedicated to measurements by means of a button system that triggers the measurement ensured the repeatability of the procedure, which included 13 test procedures (in this article, only 4 selected ones are presented). Analyses were also carried out with the use of dedicated software designed in cooperation with medical staff. It enables cataloging the list of patients (left) and easy access to signals recorded during individual tests (left middle) (Figure 5). On the right, time graphs have been included.

In the upper graph, the medical staff marks the area (movable vertical black markers) that is being analyzed. The middle graph shows the signal fragment selected for analysis. Moreover, the red and blue vertical lines mark the areas for which the rms value was the highest; therefore, the appropriate analyses were performed (the details of the analyses are described later in the paper).

### 2.3. Parameterization

All analyses (vibration and sEMG) were performed identically for the left- and right-side signals. For the analysis of vibration signals, the signal was first subjected to high-pass filtration with a cut-off frequency of 50 Hz (Butterworth 3rd order). The vector signal was calculated from the triaxial acceleration signal. It was used to calculate the rms value from the 0.5 s time window shifted continuously (every one sample). Then the algorithm determined the window for which the maximum rms value was chosen. More specifically, energetic factors (rms, mean, and crest factor) were calculated from the acceleration modulus for all three directions (X, Y, and Z). The spectral parameters (M0, M1, M2, skewness, and kurtosis) were calculated from the modulus of the frequency spectral (Fourier transform) for all three directions (X, Y, and Z).

All vibration signals from the triaxial accelerometer (left- and right-side signals) were used to calculate the resultant signal accordingly (1):

(1) $$v\left[n\right]=\sqrt{{\left(x\left[n\right]\right)}^{2}+{\left(y\left[n\right]\right)}^{2}+{\left(z\left[n\right]\right)}^{2}},$$

This procedure was aimed at eliminating errors related to the full repeatability of mounting the sensors (with a headband) on the subject’s head. Further analyses concerned the investigation of the *v*[*n*] signal.

It was decided to examine the following local parameters of the vibration signals *v*[*n*] in the selected frame.

Peak value:

(2) $$\mathrm{PEAK}=v\left[n\right],$$

Root mean square:

(3) $$\mathrm{RMS}=\sqrt{\frac{1}{N}\sum_{n=1}^{N}\;v^{2}\left[n\right]},$$

Crest factor:

(4) $$\mathrm{CREST}=\frac{PEAK}{RMS},$$

Spectral moments of the 1st and 2nd order (*M*_1_, *M*_2_) for the whole frequency band (0.5 Hz to 3 kHz) were normalized (to *M*_0_).

The spectral moment of the *m*-th order is described by a general relation (5):

(5) $$M_{m}=\sum_{i=f_{0}}^{f_{N}}\;\left|S_{n}\left(f_{i}\right)\right|\cdot {\left[f_{i}\right]}^{m},$$

where *S_n_(f)* is the frequency spectrum of the *n*-th data record, *f_i_* is the midpoint frequency for the *i*-th frequency band defined for the spectral analysis, *f*_0_ is the lower band frequency, and *f_N_* is the upper band frequency.

The spectral moment of the 0-th order *M*_0_ is used for normalization of the higher order moments and is given by the following relation:

(6) $$M_{0}=\sum_{i=f_{0}}^{f_{N}}\;\left|S_{n}\left(f_{i}\right)\right|,$$

It is usually convenient to use spectral moments normalized according to relation:

(7) $$M_{m}^{*}=\frac{M_{m}}{M_{0}},$$

The moment (8) can be interpreted as a spectrum’s center of gravity (weighted average frequency):

(8) $$M_{1}^{*}=\frac{\sum_{i=f_{0}}^{f_{N}}\;\left|S_{n}\left(f_{i}\right)\right|\cdot f_{i}}{M_{0}},$$

The moment (9) can be interpreted as the spectrum dispersion around the spectrum’s center of gravity:

(9) $$M_{2}^{*}=\sqrt{\frac{\sum_{i=f_{0}}^{f_{N}}\;\left|S_{n}\left(f_{i}\right)\right|\cdot f_{i}^{2}}{M_{0}}-{\left(M_{1}^{*}\right)}^{2}},$$

Spectrum asymmetry measure parameter—skewness:

(10) $$\mathrm{SKEWNESS}=\frac{M_{3}^{*}}{{\left(M_{2}^{*}\right)}^{3}},$$

Spectrum flattening measure parameter—kurtosis:

(11) $$\mathrm{KURTOSIS}=\frac{M_{4}^{*}}{{\left(M_{2}^{*}\right)}^{2}},$$

The selection of the vibration signal parameters was related to the nature of the examined diagnostic problem: both in the time domain and in the frequency domain. The rms, peak, and crest factor parameters give information about the energy carried by the working joint. The frequency parameters *M*_0_, *M*_1_, and *M*_2_ inform the distribution of frequency components: the center of gravity of the spectrum and the measure of dispersion from this center. On the other hand, the skewness parameter informs the asymmetry of the spectrum shape; if it is clearly different from 0, then the examined spectrum shape is asymmetrical (for comparison, the normal distribution is symmetrical). If the kurtosis parameter is clearly different from 0, then the examined shape of the spectrum is either more flattened or slender compared to the normal distribution. The proposed parameterization will serve as input data for the development of statistics in at least three stages of rehabilitation of patients with temporomandibular joint disorders. This type of approach was used in earlier research [1,46,47] relating to technical or medical assessments and diagnostics.

Electromyography (sEMG) time signals are very difficult to interpret. Therefore, there are many approaches to representing them in the literature. In the evaluation of muscle activity, studies show that better results are obtained with the energy approach. On the other hand, frequency analyses are also used (often in the analysis of muscle fatigue) [48,49,50]. Some of the most common parameters are rms (root mean square) and the mean of the absolute value of the sEMG signal [49,51]. Therefore, these two parameters were used in this study. The sEMG signal level is influenced by a number of factors, such as the psychophysical state (including skin conductivity) or the quality of skin preparation [52]. Consequently, a comparison of signals recorded on different days cannot be used. Therefore, the values are used in relation to the value obtained during the maximum contraction or the so-called MVC (maximum voluntary contraction) [53]. In our case, it was the maximum compression of the jaw without and with rollers placed between the molars. The test was performed without rollers in order to find clicks during maximum intercuspation and to assess maximum tension of the masseter muscles and, subsequently, was performed with rollers placed between the molars to assess masseter muscle tone and find how the deprogramming of the muscles changes the click or crepitation of the TMJ.

Additionally, frequency analyses were performed identical to those performed for the vibration acceleration signal. This allowed for comparative analyses between vibration signals and sEMG.

In the same was as the sEMG measurements, for acceleration, a 0.5 s time window shifted continuously (every one sample) in order to find the maximum rms values was used. That window served to obtain rms values and the mean of the absolute values as well as spectral parameters.

### 2.4. State Recognition

Using the presented measurement system, a study was conducted on a group of fifteen people (ten women and five men). Ten of the subjects were classified by the doctor as patients with TMD (unhealthy people) and the remaining five as healthy. The mean age of all people was 29.6 (±9.63) years (minimum and maximum age was 20 and 43 years). All of the unhealthy people had temporomandibular joint crackles. Eight of them were accompanied by crepitations and bruxism and seven had headaches associated with TMD. Two unhealthy people were also diagnosed with polyarticular flaccidity and one unhealthy person had limited range of joint mobility.

In the research material of this paper, we can distinguish the division of subjects into two groups: healthy people and unhealthy people. These subjects were described by a feature vector, obtained as a result of parameterization of the signal, which is an element of the k-dimensional feature space. Using the k-nearest neighbors (k-NN) method, it was decided to test the effectiveness of automatic classification of people into healthy and unhealthy.

The k-NN method is a minimum-distance method. This means that it checks the distance between the tested vector and the patterns of a given class in the feature space. Such a classifier may use different types of metrics, such as Euclidean or Chebyshev [54]. The task of the k-NN classifier is to check for which class the number of elements in the set of k closest objects to the classified vector is the largest. The distance d between objects in the most frequently used Euclidean metric is expressed is the relation:

(12) $$d\left(x,y\right)=\sqrt{\overset{N-1}{\underset{i=1}{\sum }}{\left(x_{i}-y_{i}\right)}^{2}}$$

where *N*—number of dimensions of the feature vector and *x*, *y*—analyzed feature vectors.

## 3. Results

### 3.1. Rehabilitation Progress

The results of the proposed system are shown in an example study performed in three stages of treatment of one patient. The following set of results includes: comparisons for four tests performed during three periods (the 1st before therapy; the 2nd at an early control visit, i.e., 3 months after therapy and before the pandemic; and the 3rd at 14 months after therapy and during the pandemic). The tests performed include slow opening (SO) three times, fast opening (FO) three times in each direction, opening with protruding (OP) the mandible and returning to the rest position, and slow protruding (SP) three times. Such a presentation of the results is intended to show the potential of monitoring the progress of treatment and rehabilitation of patients suffering from TMD.

**Figure 6:**
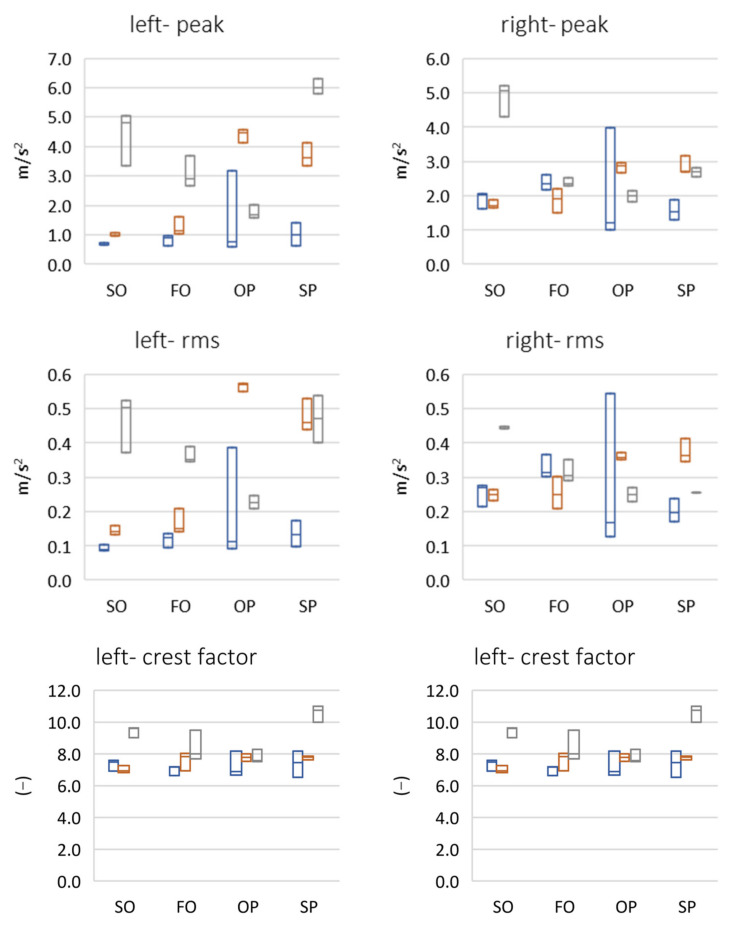
Time domain results of vibration signals (**left** and **right** side)—energy parameters (max-median-min). SO (slow opening), FO (fast opening), OP (opening with protruding), SP (slow protruding).

The analysis of peak and rms parameters indicate that the right temporomandibular joint was more active than the left one (Figure 6). In the case of both fast and slow protrusion abduction, a linear increase in the value of these parameters was detected in subsequent follow-up examinations. In an attempt to abduct from protrusion, there was a large increase in the amplitude of vibrations between the first and second period, which may indicate unblocking of the articular disc as a result of using the reposition and relaxation splint, and then a decrease for the third period due to the reduction in the frequency of physiotherapeutic procedures caused by the COVID-19 pandemic. For protrusion, there was a slow, relatively large increase in the amplitude of vibrations between periods 1 and 2, followed by another amplitude increase in the late control visit (period 3), which may have been related to the limited possibilities of physiotherapy and splint therapy sessions due to the COVID-19 pandemic. The analysis of the crest factor parameter showed an increase in its value in the 3rd period in relation to the 1st and 2nd periods, where it was comparable. There was a greater than 10-fold difference in the peak rms value in the 3rd period. This directly suggests a correlation between an increase in dynamics (range) between the peak value of the click and the remaining work energy of the joint. The analysis of the spectral moments showed that the center of gravity of the spectrum decreased for the left side, from approximately 800 Hz to approximately 200 Hz. However, for the right side, it was approximately 200 Hz.

**Figure 7:**
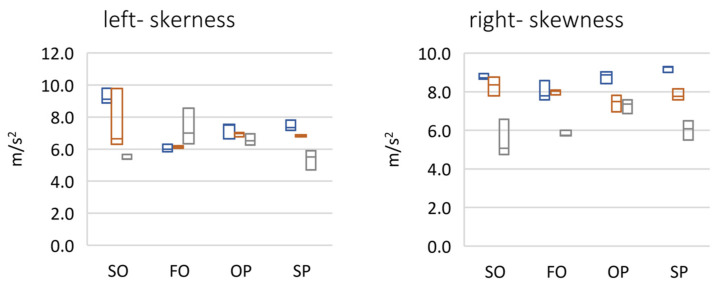

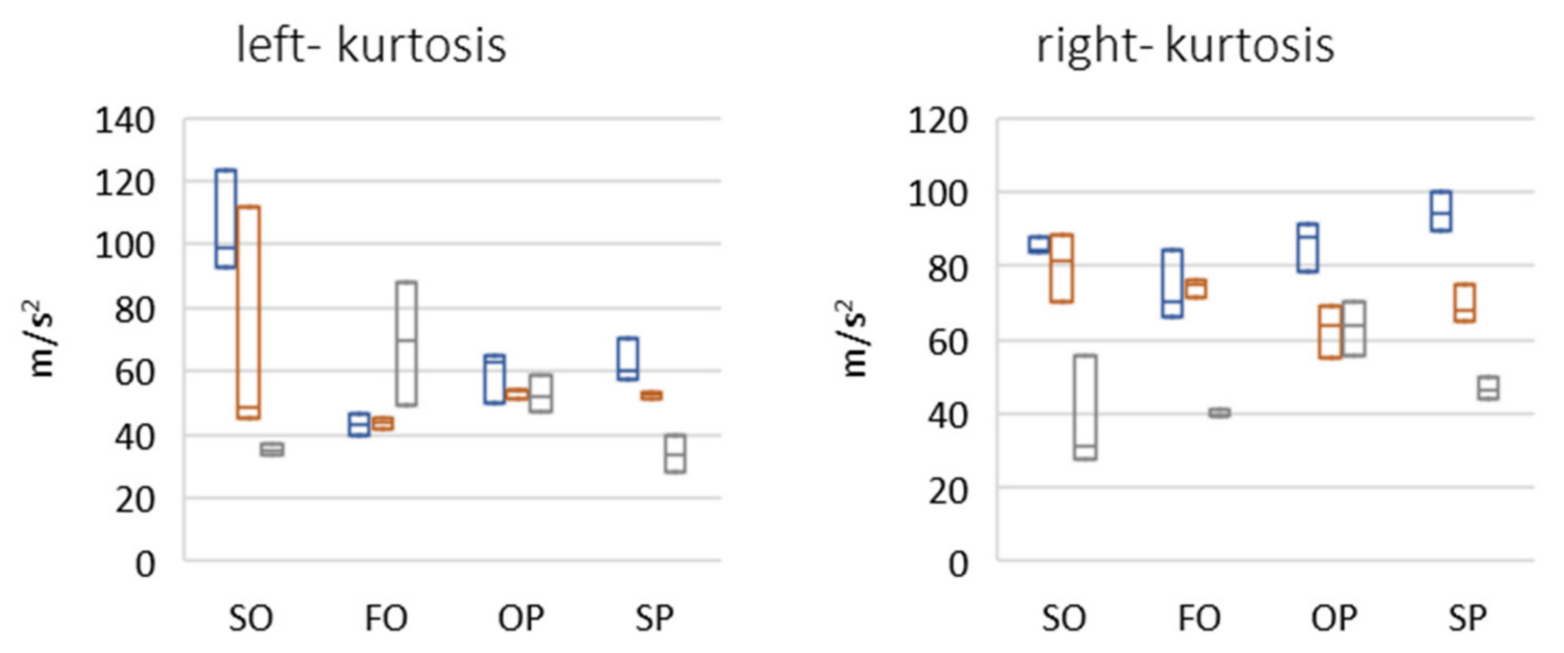
Frequency domain results of vibration signals (**left** and **right** side)—spectral shape parameters (max-median-min). SO (slow opening), FO (fast opening), OP (opening with protruding), SP (slow protruding).

**Figure 8:**
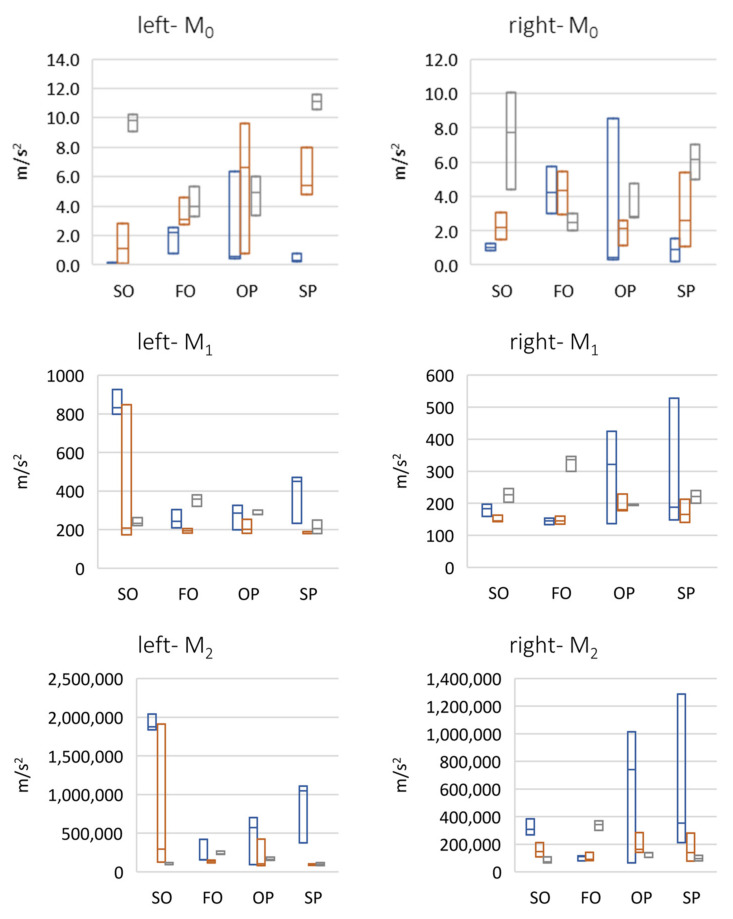
Frequency domain results of vibration signals (left and right side)—spectral moments (max-median-min). SO (slow opening), FO (fast opening), OP (opening with protruding), SP (slow protruding).

**Figure 9:**
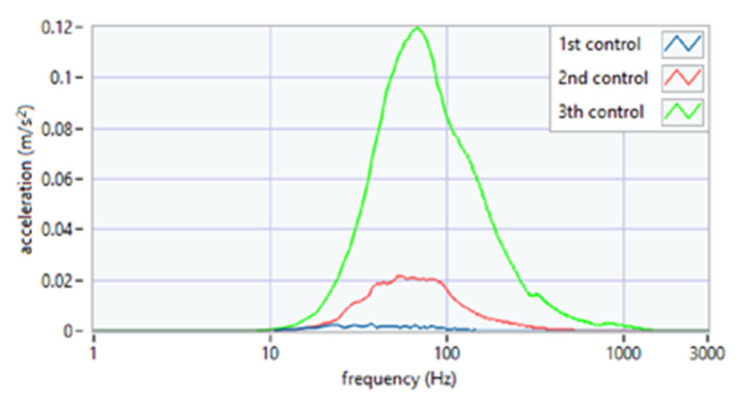
Average spectrum of slow opening (SO)—left side: 1st, 2nd, 3rd period.

**Figure 10:**
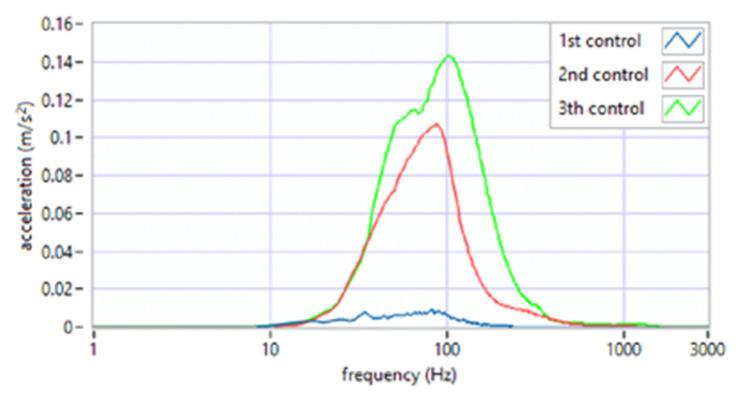
Average spectrum of slow protruding (SP)—left side: 1st, 2nd, 3rd period.

The analysis of the parameter of spectral width square depicted the nature of the click (Figure 7). Its high value suggests the flat nature of the spectrum, i.e., a crack that resembles a distinct impulse. The unit jump spectrum was perfectly linear. As can be seen from the data presented in Figure 8, a clear impulse (click) during the movement of the joint in the case of the first period was detected bilaterally. This supports the hypothesis that there is an effect related to activation (unblocking of the articular disc after the 1st period (during regular controlled splint therapy and physiotherapy). This fact was further confirmed by the spectral analysis presented in Figure 9 and Figure 10. The values of the parameter in three subsequent tests decrease by more than 20 times. An analysis of the spectrum shape parameters (skewness and kurtosis) seems to be justified only when studying a representative control group and the full group of patients.

In the case of muscle activity testing by sEMG, the MVC parameter was used (computed as a reference value to the maximum value of the determined value for each measurement series). For the patient’s left side for all performed test exercises (SO, FO, OP, and SP) an increase in the MVC value between periods 1 and 2 (controlled therapy and exercise of the patient) and a decrease between periods 2 and 3 (lack of therapy and exercise due to the COVID-19 pandemic) could be noticed. The MVC changes can be interpreted in two ways:

- Periods 1 and 2: the muscle worked at the same level of activity during the exercise but a lower maximum value was obtained during tooth clenching (as a result of muscle relaxation due to regular splint therapy and physiotherapy).
- Periods 2 and 3: there was an increase in muscle activity during maximum contraction (higher maximum value) thus a relative decrease in muscle involvement in the exercise. The reason may have been the lack of availability of physiotherapy and limitations related to splint therapy during the COVID-19 pandemic.

**Figure 11:**
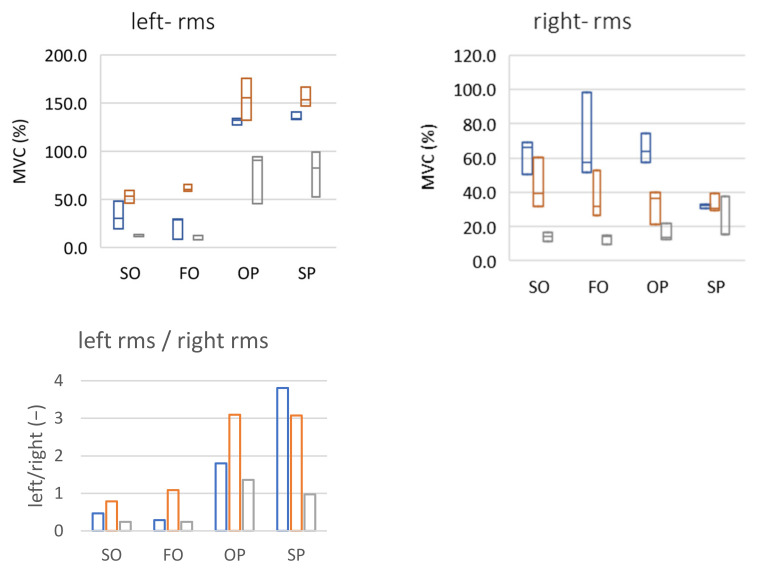
MVC of sEMG signals. Top row: left and right side (max-median-min); bottom row: the quotient of the left and right sides (mean). SO (slow opening), FO (fast opening), OP (opening with protruding), SP (slow protruding).

In the case of the signal representing the sEMG muscle activity on the right side, the changes were not as consistent: fluctuations of the obtained values oscillated around 20–30% of the MVC for SO, FO, and OP exercises for all three periods. The exception was the SP exercise for which the recorded value increased during the periods, which was most likely related to the reconstruction of the articular disc due to the difficulty in translating into the articular tubercle. The graphs illustrating the discussed phenomena are presented Figure 11.

A different approach may be an interesting observation. It seems reasonable to assume that in a properly working temporomandibular system, the load on both sides of the left and right sides should be symmetrical, and the differences should certainly not be significant. In the analyzed case, symmetry is indicated by the bottom graph (Figure 11). A value close to 1 is an ideal situation (the left and right joints are loaded identically). Any number less than 1.0 means the dominance (greater load) of the right joint, a value greater than 1.0 signifies the dominance of the left joint. Looking at both sides in this way, it can be noticed that, for SO and FO exercises, there was a change in the direction of symmetry between the first and second periods (as expected). A lack of therapy and exercise between periods 2 and 3 led to a return to dysfunction of the joints, putting more stress on the right joint. In the case of the other two exercises, the results are inconclusive (unfavorable changes for OP and a continuous decrease for SP).

The individual results of the measurements of the maximum voluntary contraction were lower each time during subsequent periods (left side: 1 = 272 μV, 2 = 210 μV, 3 = 195 μV; right side: I = 243 μV, II = 137 μV, III = 56 μV). Based on the research [53], which showed a very high variability between sEMG measurements at intervals of days, it did not necessarily indicate a gradual weakening of the muscle because too many factors affected the level of the measured signals. However, it allowed for the visualization of changes taking place in the signal and may be useful for the initial selection of the parameters of the conditioning system.

On the practical side, the procedure of designating MVC (maximum contraction value) of the examined muscles may turn out to be crucial, influencing the obtained test results. It is worth noting that during the studies conducted thus far (including unpublished results of several patients), it often happened that the MVC occurred during different exercises (e.g., for the left side while clenching without rollers and for the right side while clenching with rollers). This may be due to the fact that the rollers deprogram the muscles of the masticatory system, preventing the possibility of maximum intercuspation and maximum contraction of the masseter muscles. Therefore, it is advisable to perform both tests (with and without rollers) each time in order to obtain a reliable maximum value.

Vibration (acc) and electromyographic (EMG) phenomena during the tests (SO, FO, OP, and SP) should be related to each other. For example, muscle activity (EMG) may increase due to the force that must be exercised to overcome the resistance of the disc with a simultaneous strong vibration impulse (acc) at the time of overcoming them and translation in the joint. Therefore, a comparative analysis of the obtained results for these two types of signals was performed. These analyses were carried out separately for each of the tested tasks as each of them activated the masticatory muscles and TMJ movements in a different way. It should be noted that when comparing the repeatability of these two types of signals, the EMG signals are characterized by a greater dispersion when the patient performs the same task (test exercise). The fact that not each of the tasks generated characteristic vibration waveforms meant that, in a few cases, a similar phenomenon was observed in the change of the vibration level and muscle load.

**Figure 12:**
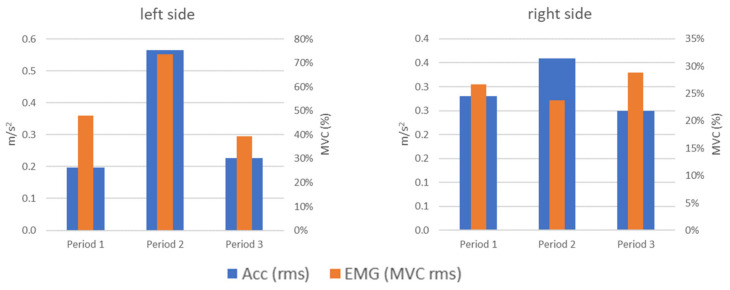
Comparison of acceleration and EMG signals for OP (opening with protruding).

An example of a compliance of parameter changes was the left side for the OP test (opening with protruding) (Figure 12). In this test, similar changes in the level of vibrations generated by the joint and the load on the muscles could be observed, which could have been caused by the partial blockage of the disc in this joint in the initial clinical examination (increase and then decrease). These changes do not coincide any more for the right side. For period 2, we see an vibration increase compared to period 1, with a simultaneous muscle activity decrease.

We can assume that a properly working joint should be characterized by symmetry both in the case of vibrations and muscle load. A comparative analysis of both sides was performed by introducing the index calculated as the quotient of the signal level recorded for the left side to the signal level recorded on the right side. A value close to 1 is an ideal situation (the left and right joints are loaded identically). Any number less than 1.0 means the dominance (greater value) of the right joint, a value greater than 1.0 signifies the dominance of the left joint.

**Figure 13:**
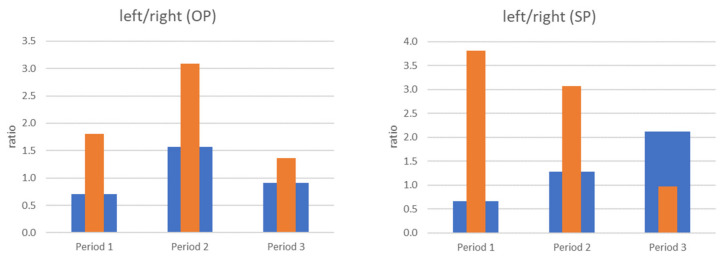
Acceleration (blue bar) and EMG signals (orange bar) ratio for OP (opening with protruding) and SP (slow protruding).

While in the case of OP (opening with protruding) there are significant differences in terms of values between the vibration and the load on the muscles (EMG), the changes taking place during the visits or periods are consistent. For Period 2, there is a noticeable increase in the percentage of the left side compared to the previous state (Period 1), and then a decrease to the level close to the baseline (Figure 13 left).

In the case of SP (slow protruding), the recorded signals behave inversely proportional (Figure 13 right). In the case of vibrations, the dominant role of the right side changes over the course of visits to a dominance of the left side, which may be related to the deformation of the left joint disc. The opposite situation occurs for the registered muscle load (EMG): the dominance of the left side during Period 1 (value > 1) is significantly weakened, leading to the balance between the right and left side (value close to 1) due to the conducted myotherapy in the form of splint therapy, physiotherapy, and autotherapy.

Figure 6, Figure 7, Figure 8 and Figure 11 present the change of vibration and EMG signal parameters during the rehabilitation process. In order to check the statistical significance of differences between individual stages of rehabilitation, statistical tests were performed for them (assumed *p* = 0.05). Among others, due to a small number of samples, the vast majority of tested groups were not characterized by normal distribution, so a non-parametric sign test was used (H0—no statistically significant differences between the tested groups). A test of the difference between the results for period 1 vs. period 2 and period 1 vs. period 3 for four exercises in total (SO, FO, OP, and SP) was performed. A sign test (equivalent to Student’s *t*-test) was used, which is a non-parametric test for dependent variables (the study involved the same patient).

Table 1 shows the results, where A means acceptance of hypothesis H0 (there is no statistically significant difference between the tested groups) and R means acceptance of alternative hypothesis H1 (there is statistically significant difference).

In Table 1 each parameter was compared independently in Period 1 (before treatment) vs. Period 2 (early control) and Period 1 vs. Period 3 (late control); differences of Period 2 vs. Period 3 were not investigated due to the specificity of this patient (interrupted rehabilitation under specialist care due to COVID-19). The aim was to see if the proposed parameterization of the vibration and EMG signal carried diagnostic information about the progress of rehabilitation (before and after rehabilitation). Therefore, a comparison of Period 2 vs. Period 3 would not be meaningful for this case (too long a break in contact with the specialist due to COVID-19).

The tests show that significant statistical changes between periods 1 and 2 and 1 and 3 occur for only the left side for the vibration parameters MAX, RMS, and MO. For the CF parameter, on the other hand, the difference occurred only between periods 1 and 3 (for both sides). For the M1 and M2 parameters, only between periods 1 and 2 for the left side was a statistically significant difference observed. For M1 and M2 parameters, there was no statistically significant difference between periods 1 and 2 and 1 and 3 (left side). For the MVC_RMS signal there are differences only between periods 1 and 2 for the left side.

For the right side there are only differences between periods 1 and 3 for parameters MAX, CF, SKEWNESS, and KURTOSIS.

The large variation in the above results confirms the impossibility of univocal inference based on the presented data. Studies on a larger statistical group are necessary.

### 3.2. k-NN Recognition

Using the k-nearest neighbors method, it was decided to test the effectiveness of automatic classification of the people described in Section 2.4 into healthy and unhealthy recognition. For the vibration signal, the feature vector was formed by the statistical parameters described in Section 2.3:

$$V_{nL/nR}=\langle \mathrm{EX},\;\mathrm{NUM},\;\mathrm{REC},\;\mathrm{PEAK},\;\mathrm{RMS},\;\mathrm{CREST},\;M_{0},\;M_{1},\;M_{2},\;\mathrm{SKEWNESS},\;\mathrm{KURTOSIS}\rangle$$

where *n* is the identification number according to the database of healthy and unhealthy people; *L* is the left side of the face; *R* is the right side of the face; EX is the name of the exercise, such as SO (slow opening), FO (fast opening), OP (opening with protruding), or SP (slow protruding); NUM is the exercise repetition number; and REC is the recognition of healthy or unhealthy.

Examples of vibrational signal features vector from the database are shown in Table 2.

For the EMG signal, the feature vector was formed by the pairs of MVC changes for the left and right sides:

$$E_{nL/nR}=\langle \mathrm{EX},\;\mathrm{NUM},\;\mathrm{REC},\;\mathrm{MVC}\rangle$$

where *n* is the identification number according to the database of healthy and unhealthy people; *L* is the left side of the face; *R* is the right side of the face; MVC is the maximum voluntary contraction; EX is the name of the exercise, such as SO (slow opening), FO (fast opening), OP (opening with protruding), and SP (slow protruding); NUM is the exercise repetition number; and REC is the recognition of healthy or unhealthy.

Examples of EMG signal features vector from the data base are shown in Table 3.

Table 4 and Table 5 show the results of the classification efficiency of both signal types for the four previously presented exercise types (slow opening (SO), fast opening (FO), opening with protruding (OP), and slow protruding (SP)). Table 3 contains calculations made on the vectors of common and disjointed features obtained in the vibration and EMG signal separately for each exercise. Table 5 contains variants of calculations taking into account two tests, giving the best results and the worst results in the studies presented in Table 4.

From the results presented in Table 4 and Table 5, it can be concluded that, for the feature vectors used, the classification performance ranged from 54.7% to 85.3%.

What should be noted is that the use of combining vibration and EMG parameters for exercises FO and OP improved the effectiveness of classification in relation to the recognition based separately on the vibration signal and EMG. As can be seen from the results presented in Table 4, it is advisable to use the classification based on these two exercises, fast opening (FO) and opening with protruding (OP), and to jointly use the vibration and EMG parameters. The expected result of the classification is at a level of 85.9%

The obtained values are not high; therefore, further work is being planned to acquire research material (further measurements of patients) and to test estimators that allow for the determination of new feature vectors.

## 4. Discussion

Temporomandibular joint disorders are a growing health problem in contemporary society and include a group of complaints related to pain in the muscles of mastication, headaches, abnormalities of mandibular movements, and pain and sound phenomena generated in the joints during mandibular movements. The classic diagnosis of temporomandibular joint dysfunction is based on imaging evaluation—X-ray, magnetic resonance, or computed tomography—performed by radiology specialists.

This paper presents the results of research conducted on the objective and non-invasive assessment of temporomandibular joint function using surface electromyography (sEMG) and analysis of vibrations generated during mandibular movement. A measurement system was developed for the purpose of the study, consisting of a hardware module for the acquisition of vibration accelerations and sEMG signals, along with proprietary software for analysis and evaluation of these signals in correlation to video images recorded within the mandible during a protocol-determined test. Few attempts to use temporomandibular joint function assessments (especially locking of the articular disk of the temporomandibular joint) based on acoustic, vibration, or EMG signal have been presented in diagnoses and evaluation of rehabilitation treatment [16,17,18,19,27,30,55,56,57]. However, the literature reports do not indicate the combination of these methods along with a new parametric assessment approach [25,29,45,58]. Several studies [59,60,61] have concluded that the clinical use of the sEMG method in the diagnosis of TMD has limited value and reported problems with the reliability and reproducibility of sEMG. In our opinion, combining the vibroacoustic method with sEMG seems to be one of the most objective, noninvasive methods to analyze and monitor TMD treatment in terms of displacement of the articular disc. The synchronous combination of measurements gives a more reliable assessment of mandibular activity and function. The limitation of using sEMG is the difficulty of assessing muscle activity after the application of immediate physiotherapy, which would require the removal of the electrodes, performing the therapy, and then replacing them, which may involve the exclusion of the area of electrode application before the therapy. The first preliminary analyses of the application of both types of signals do not allow for unambiguous assessment of their usefulness.

The k-nearest neighbor method for analyzing the parameters separately yielded results ranging from 62.5% (a low result that does not allow the clustering to be used as a method in practice) to 82.1% (an acceptable result for screening diagnostics). It is promising that, in the case of building a classifier based on both types of signals (vibration and EMG), an improvement in classification performance was obtained in some of the completed tests. An example is the opening with protruding (OP) exercise, where for each of the signals analyzed independently, 71.1% and 75.0% effectiveness were obtained, respectively, while the combined classification allowed an increase of 79.1% effectiveness. In addition, for fast opening (FO) the recognition efficiency after data fusion increased by several percent (from 73.6% to 85.3%). For the remaining exercises (slow opening (SO) and slow protruding (SP)), the results were not as promising. Thus, this indicates a significant variation in the information contained in the signals from different exercises.

Interesting results were also obtained for classification using parameters from two types of exercises performed simultaneously (Table 2). This procedure allowed the increase in the effectiveness of classification compared to the results obtained for each of the exercises separately. It is worth noting, however, that this increase was not spectacular. For example, in the case of fast opening (FO) and opening with protruding (OP) exercises, a slight increase in efficiency was obtained for both separate analysis of acceleration and EMG and for the analysis performed using both types of signal simultaneously. Nevertheless, on the basis of the analyses performed, it can be concluded that it is advisable to use claudication based on two (or more) exercises in order to increase the classification efficiency.

In conclusion, the presented results show that the use of signals from fast opening (FO) and opening with protruding (OP) exercises allows us to obtain the highest classification efficiency. The higher effectiveness of fast exercises in comparison to slow ones may be associated with greater muscle activation during their execution. The faster execution of a movement is associated with a shorter execution time and therefore greater muscle activation in a shorter period of time. This facilitates the analysis and separation of the EMG signal from the resting state (for some slow exercises this difference was very small). In addition, simultaneous relative classifiers from vibration and EMG signals together allow an increase in the classification efficiency. Furthermore, combining the classifiers from both exercises as well as from the signals (vibration and EMG) allows for a slight increase in the effectiveness of the applied classification method.

The vibration description and EMG signal parameters used in the presented analyses were chosen based on the authors’ experience. In this study, they were all used as a feature vector for the k-NN method. In the future, in order to minimize the feature vector, additional analyses should be performed to exclude features that carry the same or redundant information (for this purpose, among others, correlation techniques can be used).

It should be emphasized that the presented classification results were determined for k = 1. This is important because, in many cases, the application of the k-NN method for a larger number of k gives better results. Therefore, this is an element that will certainly be taken into account in future research. Additionally, we assume that effectiveness can also be increased with the use of other classifiers and by increasing the amount of data to be analyzed.

A practical application of this study is presenting to the patient the correlation between excessive muscle tension and acoustic sensations, which may contribute to better awareness of the problem and implementation of early diagnosis and prevention of serious consequences such as blockage of the TMJ disc as well as degenerative changes on the surfaces of the temporomandibular joints. It can help in justifying the importance of physioprophylaxis of TMD disorders and implementing rehabilitation as early as possible. However, further research on outcomes, safety, and long-term quality of life data with the application of randomized controlled trials is recommended. Such research is currently being conducted by the authors.

### Limitations

The main limitation in inference was the small statistical group. The authors are in the process of collecting a large statistical group of TMD sufferers and non-TMD sufferers so that a strong statistical analysis of the obtained research results is possible. In particular, we aim to assess the validity of the assumed feature space, including feature correlations and thus redundancy of information. Another limitation was showing the results for only one classification method: k-NN. The results obtained so far are promising and indicate the validity of other methods. The authors would like to emphasize that the purpose of this paper was to show the possibility of combining several measurement techniques to evaluate TMD.

## Figures and Tables

**Table 1:** Statistical significance of differences between the individual stages of rehabilitation (R—there is a difference, A—there is no difference).

		Left Side		Right Side	
		Period			
		1–2	1–3	1–2	1–3
VIB	MAX	R	R	A	R
	RMS	R	R	A	A
	CF	A	R	A	R
	M0	R	R	A	A
	M1	R	A	A	A
	M2	R	A	A	A
	SKEWNESS	A	A	A	R
	KUROSIS	A	A	A	R
EMG	MVC_RMS	R	A	A	A

**Table 2:** Examples of vibrational signal features vector.

	EX	NUM	REC	PEAK	RMS	CREST	M0	M1	M2	Skewness	Kurtosis
V_1L_	SO	2	uh	7.3	1.2	5.9	7.1	637	1,176,830	5.8	39.4
V_1R_	SO	2	uh	13.8	2.3	6.1	5.0	211	102,890	6.1	46.2
V_1L_	FO	2	uh	8.8	1.0	8.5	3.5	302	211,548	5.2	33.9
V_1R_	FO	2	uh	1.8	0.3	5.8	1.7	321	345,269	5.9	40.8
V_1L_	OP	2	uh	0.1	0.02	4.5	0.1	574	1,295,310	8.3	74.4
V_1R_	OP	2	uh	3.2	0.3	11.6	1.5	463	442,047	3.7	19.2
V_1L_	SP	2	uh	0.03	0.01	3.1	0.06	807	1,670,840	11.3	164.4
V_1R_	SP	2	uh	0.05	0.01	3.6	0.04	995	214,817	8.9	89.8
V_11L_	SO	3	h	0.07	0.01	6.8	0.04	1329	2,495,700	10.9	144.7
V_11R_	SO	3	h	0.04	0.01	2.9	0.04	1374.3	2,525,430	8.4	82.0
V_11L_	FO	3	h	0.03	0.1	2.8	0.03	1362.9	2.51 × 10^6^	13.5	218.4
V_11R_	FO	3	h	0.16	0.03	4.7	0.24	453.7	743,576	6.14	49.6
V_11L_	OP	3	h	0.06	0.01	3.8	0.04	869.4	1.96 × 10^6^	10.2	116.7
V_11R_	OP	3	h	0.1	0.02	4.7	0.2	424.3	1.0 × 10^6^	9.8	111.8
V_11L_	SP	3	h	0.02	0.01	2.0	0.02	1353.8	2.5 × 10^6^	12.7	190.1
V_11R_	SP	3	h	0.03	0.01	2.6	0.05	972.9	2.07 × 10^6^	11.1	141.7

**Table 3:** Examples of EMG features vector.

	EX	NUM	REC	MVC
E_1L_	SO	2	uh	11.51
E_1R_	SO	2	uh	2.90
E_1L_	FO	2	uh	12.60
E_1R_	FO	2	uh	3.31
E_1L_	OP	2	uh	43.69
E_1R_	OP	2	uh	11.28
E_1L_	SP	2	uh	40.68
E_1R_	SP	2	uh	9.75
E_11L_	SO	3	h	12.71
E_11R_	SO	3	h	7.67
E_11L_	FO	3	h	12.38
E_11R_	FO	3	h	7.50
E_11L_	OP	3	h	26.03
E_11R_	OP	3	h	10.32
E_11L_	SP	3	h	24.44
E_11R_	SP	3	h	6.63

**Table 4:** Effectiveness of k-NN classification.

Effectiveness of Classification [%]							
Slow Opening (SO)		Fast Opening (FO)		Opening with Protruding (OP)		Slow Protruding (SP)	
VIB	EMG	VIB	EMG	VIB	EMG	VIB	EMG
76.6	62.5	82.1	68.7	71.1	75.0	73.6	62.5
VIB + EMG		VIB + EMG		VIB + EMG		VIB + EMG	
62.1		85.3		79.1		56.3	

**Table 5:** Effectiveness of k-NN classification.

Effectiveness of Classification [%]			
Slow Opening (SO) + Slow Protruding (SP)		Fast Opening (FO) + Opening with Protruding (OP)	
VIB	EMG	VIB	EMG
79.8	54.7	84.3	63.3
VIB + EMG		VIB + EMG	
63.6		85.9	

## Data Availability

Not applicable.
